# Thermoelectric Property Mapping for High‐Performance Integrated MgAgSb‐MgCuSb System

**DOI:** 10.1002/advs.202520889

**Published:** 2026-01-15

**Authors:** Jiankang Li, Airan Li, Longquan Wang, Xinzhi Wu, Raju Chetty, Takao Mori

**Affiliations:** ^1^ Research Center for Materials Nanoarchitectonics (MANA) National Institute for Materials Science (NIMS) Tsukuba Japan; ^2^ Graduate School of Pure and Applied Sciences University of Tsukuba Tsukuba Japan

**Keywords:** high‐efficiency, interface, MgAgSb, MgCuSb, thermoelectric

## Abstract

To achieve high conversion efficiency in thermoelectric (TE) modules, not only thermoelectric materials (TEMs) with high figure‐of‐merit *zT* but also the optimization of thermoelectric interface materials (TEiMs) is required. For TEMs, enhancing *zT* relies on increasing power factor PF and lowering thermal conductivity *κ*, while for TEiMs, high electrical conductivity and *κ* are essential, which highlights the importance of the integrated design of TEMs and TEiMs. In this work, we select p‐type MgAgSb as the object and construct a TE property mapping of the integrated MgAgSb–MgCuSb two‐phase system by tuning the Ag/Cu ratio. Based on the thermoelectric property mapping, Ag‐rich compositions exhibit superior PF and *zT* values for TEM, maximized in PF∼21 µW cm^−1^ K^−2^, *zT* = 1.12 for MgAg_0.97_Cu_0.03_Sb. Conversely, Cu‐rich composition MgAg_0.05_Cu_0.95_Sb is identified as the optimal TEiM for its low contact resistance and superior carrier and phonon transport properties, reducing energy loss. A two‐pair thermoelectric module integrated with n‐type Mg_3_(Sb, Bi)_2_ is successfully fabricated, yielding a peak conversion efficiency of ∼7.2%, thereby advancing the performance of current Mg‐based TE modules. Overall, this study realizes the synergistic optimization of high‐performance TE materials and compatible TE interface materials, paving the way for the fabrication of efficient and scalable TE modules.

## Graphical abstract

From the property mapping of the MgAgSb–MgCuSb system, both thermoelectric materials and corresponding interface materials are optimized: Ag‐rich compositions provide higher PF and *zT*, whereas Cu‐rich side yields superior transport properties and low contact resistance. A two‐pair module is also fabricated with n‐type Mg_3_Bi_1.5_Sb_0.5_, and it reaches an efficiency of ∼7.2% at Δ*T* = 293 K, demonstrating an effective strategy for high‐performance Mg‐based TE modules.

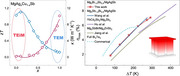

## 1 Introduction

A significant portion of energy—around 60%—used in sectors like steelmaking, chemicals, light manufacturing, and food processing ends up lost as waste heat [1, 2]. Capturing and repurposing this thermal energy more effectively is essential for enhancing overall energy utilization, reducing dependence on finite energy resources, supporting carbon neutrality goals, and addressing the urgent challenges of climate change [3]. In this scenario, thermoelectric generators (TEGs), which enable the direct conversion of heat energy into electricity based on the Seebeck effect, offer a promising technology for recycling wasted heat energy [4, 5, 6]. With the promotion of global industrialization, which requires a larger scale of operations in pursuit of higher waste energy conversion efficiency, the significance of advancing and upgrading existing thermoelectric (TE) devices is underscored [7].

TE modules consist of the following components, including cold side and hot side substrates with electrode circuits, p‐type and n‐type TE materials, and their corresponding TE interface materials (TEiMs) [8]. The conversion efficiency (*η*) of TE devices is positively correlated with the TE performance of p‐ and n‐type materials and the selection of suitable TEiMs [9, 10]. The *η* for TE single leg can be described with the equation $\eta =\frac{T_{h}-T_{c}}{2T_{h}}\left(\frac{1}{2-\frac{T_{h}-T_{c}}{2T_{h}}+\frac{4}{zT_{h}}\left(1+\frac{\lambda }{L}\right)}\right)\;$[11, 12], where *T*_h_ and *T*_h_ represent hot side temperature and cold side temperature, respectively, *zT*_h_ represents the *zT* value in the hot side, and *L* represents the length of TE legs. The *λ* is the interfacial resistivity parameter, which is described as *λ* = 4*ρ*_c_/*ρ*, where *ρ*_c_ is the contact resistivity, and *ρ* is the electrical resistivity of TE materials. From the equation, it can be seen that the value of *zT* and *λ* directly influence the overall energy conversion efficiency of the device. Hence, a comprehensive consideration of both *zT* enhancement and *λ* reduction is essential for pursuing higher *η*, as will be discussed in detail below.

The dimensionless figure of merit (*zT*), which gauges the TE conversion performance of a material, is defined as *zT* = *S* ^2^ *σT*/ (*κ_e_* + *κ*_L_), where *S* is the Seebeck coefficient, *σ* is the electrical conductivity, *T* is the absolute temperature, and *κ*_L_ and *κ*_e_ are the lattice and electronic components of total thermal conductivity *κ*, respectively. The higher *zT* generates a higher *η*, which requires improving the power factor (PF = *S* ^2^ *σ*), synergized with the suppression of *κ*. However, the complex correlation between those parameters makes it difficult to simply improve TE performance. Various principles have emerged and been developed for the optimization of *zT*, such as band structure engineering [13], introduction of point defects [14, 15], softening strategy on crystal lattice [16, 17, 18], and entropy engineering [19].

Unlike many electronics operating near room temperature (RT), TE devices must function at elevated temperatures. This requires TEiMs, which work as a barrier layer (TEiM/TEM/TEiM), to ensure efficient heat and electron transfer between ceramic substrates and TE materials for long‐term stability, demonstrating that higher *σ* as well as *κ* are needed for a suitable TEiM [20, 21, 22, 23, 24, 25]. It is also worth noting that the interfacial resistivity parameter, *λ* (4*ρ*_c_/*ρ*), should be reduced by reducing *ρ*_c_ for higher *η*. Since a higher *σ* (i.e., lower *ρ*) is generally inevitable in the optimization of *zT*, which will increase *λ* and consequently degrade *η*, it is essential to minimize *ρ*_c_ synergistically to suppress the degradation of *η*. In conclusion, the optimization of TE modules should be pursued from an integrated perspective, involving both the enhancement of *zT* and the suppression of *ρ*_c_, as will be discussed in this work.

Right now, only Bi_2_Te_3_‐based TE devices have realized large‐scale commercialization in the mature TE technology market due to their superior efficiency near room temperature (RT) [26, 27]. However, the strong bipolar effect will result in a rapid degradation of *zT* above 373 K, posing a challenge for their application in mid to high temperature power generation [28]. Furthermore, considering the scarcity of the Tellurium (Te) element in the earth's abundance (1×10^−3^ ppm), finding a Te‐free as well as high TE performance material to construct Te‐free TEGs has become a crucial but intriguing topic [29, 30, 31]. Encouragingly, Mg‐based TEGs composed of p‐type MgAgSb and n‐type Mg_3_(Bi, Sb)_2_ have been recognized as a high‐potential candidate owing to their environment‐friendly composition as well as high TE performance from 300 to 600 K [9, 32, 33, 34, 35]. For p‐type MgAgSb, the crystal structures and TE performance are systematically revealed by Melanie et al., [36]. Several phase transitions (α‐, β‐, and γ‐phases, respectively) are observed at different temperatures, and *α*‐MgAgSb exhibits the promising TE performance in the low temperature range for its intrinsic low *κ*, proving its high potential as a p‐type component in TE modules [36, 37].

As we mentioned before, to develop high‐performance TE modules based on MgAgSb, both the intrinsic material properties and the interfacial engineering must be considered holistically [17, 38, 39, 40]. Previous research has predominantly concentrated on enhancing the TE performance of the materials or identifying appropriate TEiMs [20, 41]. A major challenge lies in the strong composition sensitivity of MgAgSb, where minor deviations from the stoichiometric ratio can lead to significant degradation in TE performance, thus complicating the reproducibility and scalability of high‐efficiency materials [39, 42]. Meanwhile, reliable TEiMs are equally critical in ensuring long‐term device stability, as interfacial deterioration and elemental interdiffusion can induce performance decay at both material and device levels. Therefore, a comprehensive understanding and integrated design of the TE junction, encompassing both the functional materials and interfacial layers, is essential for realizing efficient and scalable MgAgSb‐based TE modules. MgCuSb, which belongs to the half‐Heusler (HH) family of compounds, exhibits metallic behavior owing to the lack of a bandgap and consequently shows intrinsic inferior TE performance [43]. However, its potential both as TEiM or as a second phase to enhance the TE performance of the matrix has been reported by *Xie* et al., [20, 44]. This observation provides a valuable insight, motivating the integral design of MgAgSb‐based TE junctions via two‐phase alloying to simultaneously realize optimized TEMs and TEiMs.

In this work, we design a TE property mapping for the MgAgSb–MgCuSb two‐phase system by incorporating Cu as a secondary metallic element into the MgAgSb matrix. The in situ formed MgCuSb nanoprecipitates are homogenously formed in the MgAgSb matrix, and thus a series of MgAg*_x_* Cu_1‐_ *_x_* Sb‐based TE materials (*x* = 0 to 1) are obtained. With this TE property mapping, high‐potential TE materials and corresponding suitable TEiM can be discovered at the same time. With the 3% Cu doping into the matrix, the in situ formed MgCuSb increases the carrier concentration and optimizes the PF. As a result, a high PF of ∼21 µW cm^−1^ K^−2^ at 323 K is obtained in MgAg_0.97_Cu_0.03_Sb. The corresponding suitable TEiM is chosen as MgAg_0.05_Cu_0.95_Sb, which exhibits high *σ* and *κ*, guaranteeing less energy loss during carrier transport. The screened MgAg_0.97_Cu_0.03_Sb as TE material and MgAg_0.05_Cu_0.95_Sb as TEiM are chosen, and the corresponding TE single leg is synthesized. The advanced interface demonstrates a minimal contact resistance of ∼6.7 µΩ cm^2^ and maintains holistic stability after 2 weeks of aging. A two‐pair TE module with MgAgSb and n‐type Mg_3_(Sb, Bi)_2_ is also fabricated, and a maximum efficiency of ∼7.2% achieved, advancing the existing Mg‐based TE modules. The introduced alloying strategy guarantees the global design of high‐performance TE modules and inspires advancing the industrialization of tellurium‐free TE modules for power generation.

## 2 Results and Discussion

### 2.1 Thermoelectric Property Mapping in the Integrated MgAgSb‐MgCuSb System

**FIGURE 1:**
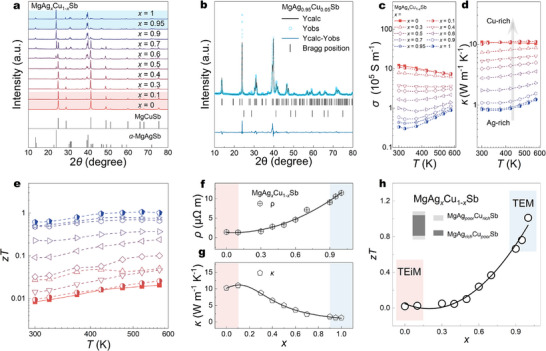
(a) XRD patterns of MgAg*_x_* Cu_1‐_ *_x_* Sb (*x* = 0–1). (b) The Rietveld refinement result of MgAg_0.95_Cu_0.05_Sb, (c–e) Temperature‐dependent (c) *σ*, (d) *κ*, (e) *zT* of MgAg*_x_* Cu_1‐_ *_x_* Sb (*x* = 0–1), (f–h) 573 K (f) *σ*, (g) *κ*, (h) *zT* property mapping of MgAg*_x_* Cu_1‐_ *_x_* Sb (*x* = 0–1).

Figure S1 presents a schematic diagram of a 2‐pair TE module, which comprises ceramic substrates, a Cu electrode, TEiM, and TE materials, indicating that the choice of TEiM and TE significantly influences the *η* of the module. To explore a better TE material with its suitable TEiM, an integrated MgAgSb‐MgCuSb system is designed. By different ratios of Cu and Ag doping into the matrix, a series of MgAg*_x_* Cu_1‐_ *_x_* Sb‐based TE materials (*x* = 0 to 1) is obtained. Figure 1a shows the patterns of X‐ray diffraction (XRD) for MgAg*_x_* Cu_1‐_ *_x_* Sb (*x* = 0 to 1). At *x* = 1, the XRD pattern is in good agreement with the standard PDF card of α‐MgAgSb, and no secondary phases or impurity peaks were observed under the limitation of detection, confirming that α‐MgAgSb is the sole dominant phase in the sample. Additionally, the X‐ray spectroscopy (EDX) pattern of α‐MgAgSb reveals that all elements are distributed homogeneously (Figure S2). With the content of Cu increased, there are two phases that can be detected from the XRD pattern. At 2*θ* = 28.6°, the (2 0 0) Bragg peaks in the MgCuSb phase show in *x* = 0.95, and (1 1 0) Bragg peaks can be detected in *x* = 0.9 at 2*θ* = 24.9°, indicating the MgCuSb phase is already formed. The high‐magnification SEM image and EDX mappings in Figure S3 also reveal the area where Cu enrichment is plentiful, and Ag is deficient. It is demonstrated that in situ formed MgCuSb is formed uniformly in the matrix, similar phenomena also have been reported by *Xie* et al., [44]. Moreover, the result of Rietveld refinement for *x* = 0.95 to quantify the phase fraction of MgCuSb is shown in Figure 1b, and the phase fraction of MgCuSb is ∼2.77%, confirming the formation of the MgCuSb phase by excess Cu doping. As the Cu content increases, the relative phase intensity of MgCuSb gradually increases, whereas that of MgAgSb weakens, suggesting a progressive substitution of Ag by Cu and a corresponding increase in MgCuSb content. This trend demonstrates a gradual phase transition from MgAgSb to MgCuSb with continuous Cu substitution. For *x* = 0.3, only two diffraction peaks at 2*θ* = 39° and 39.3°, corresponding to the (4 0 0) and (2 2 4) planes of α‐MgAgSb, can be detected, suggesting that the compound has almost completely converted to the MgCuSb phase. In the end, when *x* = 0, the corresponding XRD pattern matches the standard PDF card of MgCuSb well, and all elements are spread uniformly from the EDX pattern (Figure S4), confirming that MgCuSb is the only dominant phase.

TE performances of all samples are measured and plotted into a mapping format for a deeper understanding of the TE transport properties in the MgAgSb—MgCuSb integral system. Figure 1c,d shows the temperature dependence of *σ* and *κ*, respectively. It is important to note that for Ag‐rich samples (*x* ≥ 0.8), the *σ* and *κ* first decrease and then increase with increasing temperature. Deteriorated *σ* and *κ* should be ascribed to the inherent bipolar effect, which extensively exists in narrow‐gap semiconductors [45, 46]. It is the temperature gradient that drives both electrons and holes to move in the same direction, which results in the degradation of the total value of *S* (Figure S5), and currents of electrons and holes bring higher *σ* and *κ*. With increasing Cu content, the bipolar effect is gradually suppressed in samples containing 50% or more Cu, implying that the metallic MgCuSb phase becomes dominant in the composite. Cu‐rich samples (*x* ≤ 0.4) exhibited metallic *σ* and *κ*, which are much higher than those of Ag‐rich samples, owing to the increasing content of the metallic MgCuSb phase. At 300 K, the highest *σ* is 1.2 × 10^6^ S m^−1^ in the Cu‐rich sample (*x* = 0), almost 40 times higher than in the Ag‐rich (*σ* ∼3.3 × 10^4^ S m^−1^ of the sample *x* = 1). A similar trend can also be found in the *κ* for this binary system, the samples in Cu‐rich side (*κ* ∼10.2 W m^−1^ K^−1^ of *x* = 0) show much higher *κ* than those in Ag‐rich side (*κ* ∼1.2 W m^−1^ K^−1^ of *x* = 1), which is mainly contributed by the intrinsic high *κ*_e_ of MgCuSb (Figure S6). As a result, the *zT* of all samples is calculated, as Figure 1e shown, sample *x* = 1 exhibits the highest *zT* ∼1.02 at 473 K, while with the content of Cu increasing, the *zT* decreases gradually, the *zT* of *x* = 0 is ∼0.02 as a prominent contrast. As we emphasized before, high values of *σ* and *κ* are desired for the TEiM to minimize energy dissipation during heat and charge transport [47], whereas a high *zT* as well as low *κ* are essential for the TE material. According to Figure 1f,g, which presents the *ρ* (1/*σ*) and *κ* at 573 K for the MgAgSb–MgCuSb binary system, the samples in the Cu‐rich region (*x* = 0–0.1) exhibit higher *σ* and *κ*, indicating superior carrier and phonon transport properties and suggesting its high potential as an ideal TEiM for MgAgSb. In contrast, the samples in the Ag‐rich region (*x* = 0.95–1) show a significantly superior *zT* than the Cu‐rich region (Figure 1h), demonstrating its superior TE performance and suitability for TE material.

Based on the mapping of TE properties in this binary system, the optimal composition range for MgAgSb–MgCuSb TE junctions can be narrowed down to two regions: the Ag‐rich side (*x* = 0.97, 0.99, and 1) and the Cu‐rich side (*x* = 0–0.1). Further subdivisions within the Ag‐rich (*x* = 1, 0.99, and 0.97) and Cu‐rich (*x* = 0.05 and 0) regions have been carried out to investigate the ideal compositions with improved TE performance. It should mention that the small deviation in Ag/Cu ratios in the Ag‐rich side is hard to control, given the high composition sensitivity in this binary system. To solve this issue, 0.5% stearic acid (C_18_H_36_O_2_) is also added into the matrix (*x* = 0.97, 0.99, and 1) to reduce the powder adhesion during the ball milling process and achieve excellent repeatability of powder. Moreover, TE performance of *x* = 1 is further optimized by the weakened chemical bonds that reduce phonon velocity and *κ* [17] (Figure S7). Figure S7a shows the patterns of XRD for MgAg*_x_* Cu_1‐_ *_x_* Sb (*x* = 0.97, 0.99, and 1). In sample *x* = 0.99, the absence of the MgCuSb secondary phase in the XRD pattern suggests that Cu may be incorporated into the MgAgSb lattice. The Rietveld refinement results of MgAg*_x_* Cu_1‐_ *_x_* Sb (*x* = 0–1) demonstrate that almost no MgCuSb phase is detected in the sample *x* = 0.99 (Figure S8), indicating that the minor amount of Cu will tend to solubilize in MgAgSb [48]. With the content of Cu increasing to 3%, the matrix is saturated, and the excess Cu (∼1%) will form the MgCuSb second phase [44], as supported by the binary phase mapping drawn in Figure S9, proving the Cu solubility limit in the MgAgSb may be around 2%. A similar trend can also be found in the Cu‐rich sample. Figure S7b exhibits the patterns of XRD for MgAg*_x_* Cu_1‐_ *_x_* Sb (*x* = 0, 0.05, and 0.1). The α‐MgAgSb secondary phase is identified in the *x* = 0.1 sample, where ∼2.2% MgAgSb is formed in MgAg_0.1_​Cu_0.9_​Sb, together with additional metallic secondary phases in MgAg_0.05_​Cu_0.95​_Sb, indicating the limited solid solubility of Ag in MgCuSb, which is crucial for understanding and optimizing the TEiMs.

### 2.2 The Optimization of the Ag‐Rich Side for Ideal TE Materials

**FIGURE 2:**
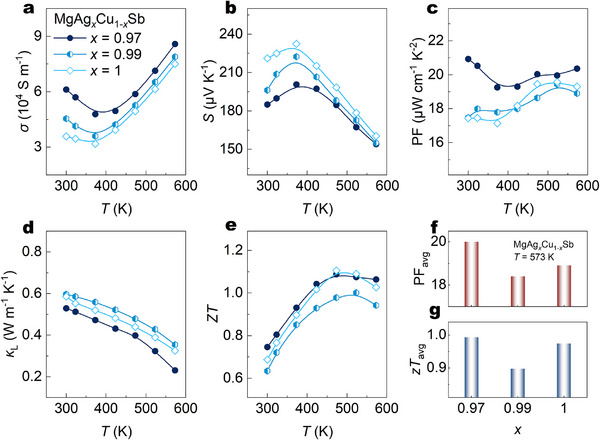
(a–h) Temperature‐dependent (a) *σ*, (b) *S*, (c) PF, (d) *κ_L_*, (e) *zT* of MgAg*_x_* Cu_1‐_ *_x_* Sb (*x* = 0.97, 0.99 and 1), (f),(g) (f) PF_avg_, (g) *zT*_avg_ of MgAg*_x_* Cu_1‐_ *_x_* Sb (*x* = 0.97, 0.99 and 1).

The temperature dependence of *σ* and *S* for MgAg*_x_* Cu_1‐_ *_x_* Sb (*x* = 1, 0.99, 0.97) samples is shown in Figure 2a,b. The gradual increase in *σ* and the corresponding decrease in *S* with increasing Cu content indicate that Cu doping leads to a higher carrier concentration (*n*_H_) in the system [49]. For sample *x* = 0.97, the *σ* at 300 K increases to 6.11 × 10^4^ S m^−1^, nearly 70% elevated compared with 3.57 × 10^4^ S m^−1^ for the pristine MgAgSb (*x* = 1). Higher *n*_H_ can be contributed by the introduction of Cu into the matrix. For the sample with *x* = 0.99, it can be speculated that the increase in *n*_H_ is associated with the reduced formation energy of Ag vacancies induced by Cu incorporation into the lattice [49]. It should be noted that for the sample *x* = 0.97, the formation of MgCuSb is detected and quantified through the Rietveld refinement discussed before (Figure S8). Therefore, the increased *n*_H_ does not only arise from Cu incorporation into Ag vacancies as in the *x* = 0.99 case, but in addition, the established Ohmic contact for MgAgSb/MgCuSb interface can also contribute [44]. Namely, with the good contact, and being driven by the chemical potential difference, electrons will move from MgAgSb to MgCuSb, and more holes are formed, increasing the *n*_H_ and *σ*, which corresponds with the previous report [44]. As a result, with *x* rising from 0 to 0.03, *n*_H_ increases from 3.95 × 10^19^ to 6.06 × 10^19^ cm^−3^ (Figure S11a). Thanks to the improvement in *n*_H_, an optimization of PF is realized after minor Cu doping due to the enhanced *σ*. As shown in Figure 2d, for MgAg_0.97_Cu_0.03_Sb, the highest PF reaches 20.9 µWcm^−1^ K^−2^ at 300 K, realizing ∼20% improvement compared with MgAgSb, and even at 573 K, the PF also displays 20.4 µWcm^−1^ K^−2^, maintaining a high performance over the whole temperature range.

For most bulk materials, *κ* can be considered to be the sum of two contributions, which are *κ*_L_ due to the phonon transport and *κ*_e_ depends on the carrier transport (electrons and holes). For *κ*_L_, since it can be expressed by the formula *κ*_L_ = 1/3 × *c*_v_ *ν*_g_ *l*, where *c*_v_ represents specific heat, *ν*_g_ phonon group velocity, and *l* phonon mean free path, which is strongly independent of other TE parameters, should be minimized as much as possible [17]. In this work, we found that the minor existence of MgCuSb can effectively suppress the phonon transport, realizing a decrease in the *κ*_L_. As Figure 2e shows, the *κ*_L_ reaches around 0.53 W m^−1^ K^−1^ at 300 K for the *x* = 0.97 sample, realizing nearly a 13% decrease compared with 0.60 W m^−1^ K^−1^ of the pristine sample. Moreover, when the temperature reaches 573 K, the *κ*_L_ of sample *x* = 0.97 decreases to 0.23 W m^−1^ K^−1^, resulting in ∼ 30% decrease in contrast with 0.32 W m^−1^ K^−1^ of the *x* = 1 sample. The decreased *κ*_L_ exhibits that it is the in situ formed MgCuSb nanoprecipitates that enhance the phonon scattering, therefore, resulting in a decrease in phonon mean free path and *κ*_L_. According to the Wiedemann‐Franz law, *κ*_e_ and *σ* are correlated and can be expressed by *κ*_e_ = *LσT*, where *L* is the Lorenz number. As Figure S11 shows, the value of *κ*_e_ increases naturally owing to the increased Cu content optimized *σ*, which is especially evident from 300 to 425 K. As proof, at 300 K, *κ*_e_ increases from ∼0.18 W m^−1^ K^−1^ (*x* = 1) to ∼0.31 W m^−1^ K^−1^ (*x* = 0.97), and *κ*_e_ at 573 K varies from 0.75 W m^−1^ K^−1^ to 0.87 W m^−1^ K^−1^.

Owing to the value of the increase in *κ*_e_ being higher than the decrease in *κ*_L_, the value of *κ* realizes a slight increase inevitably (Figure S11c). The *zT* of all samples is calculated and plotted in Figure 2h, and it is encouraging that the *zT* is optimized thanks to the increased *σ* by Cu doping. Sample *x* = 0.97 exhibits the highest *zT* value of 0.75 and 1.1 at 300 and 573 K, respectively. Average PF (PF_avg_) and Average *zT* (*zT*_avg_) are also calculated to evaluate the ability of optimized samples for practical TE conversion efficiency compared with the pristine sample. As Figure 2f shows, the enhanced PF_avg_ in the sample *x* = 0.97 is beneficial for a higher output power of the module [44]. Also, sample *x* = 0.97 realized a slightly improved *zT*_avg_ compared with the MgAgSb sample, which is beneficial for the improvement of the output efficiency, indicating that MgAg_0.97_Cu_0.03_Sb should be used over MgAgSb as TEM in the MgCuSb‐MgAgSb binary system.

### 2.3 The Optimization in Cu‐Rich Side for Ideal TEiM

**FIGURE 3:**
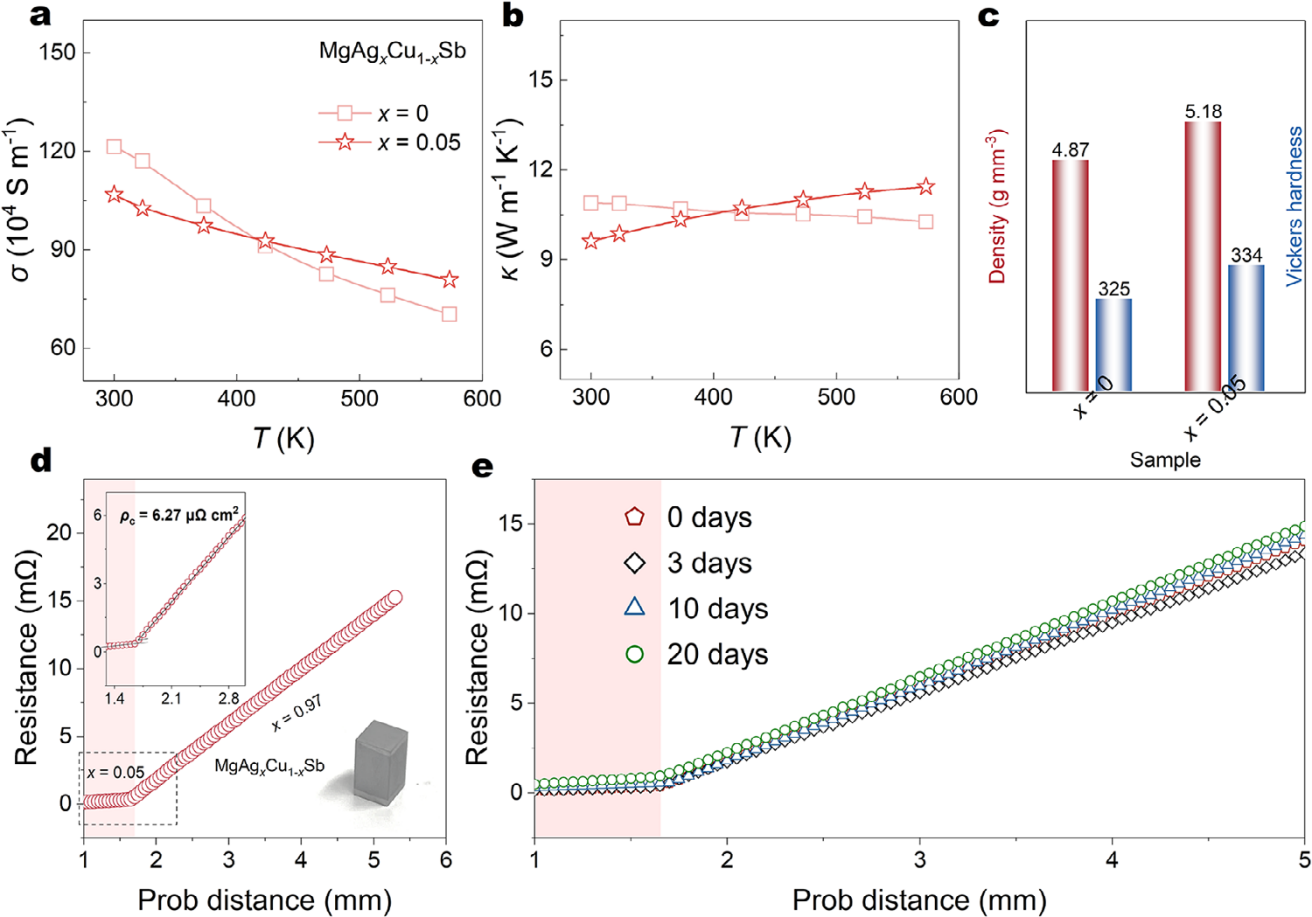
Temperature‐dependent (a) *σ*, (b) *κ* of MgAg*_x_* Cu_1‐_ *_x_* Sb (*x* = 0 and 0.05), (c) Comparation of mass density and Vickers hardness between *x* = 0 and *x* = 0.05 samples, (d) Probe distance dependence of resistance in MgAg_0.05_Cu_0.95_Sb/MgAg_0.97_Cu_0.03_Sb/MgAg_0.05_Cu_0.95_Sb TE single leg, the optical image is image of the corresponded single leg, (e) Probe distance dependence of resistance after 0–20 days aging.

To find suitable TEiM in the Cu‐rich side, we prepare *x* = 0 and *x* = 0.05 samples for comparison. All compositions maintain relatively high *σ* and *κ* values, with the *x* = 0.05 sample exhibiting higher values at elevated temperatures, outperforming the *x* = 0 sample and suggesting improved charge and heat transport properties. The other temperature‐dependent TE properties are shown in Figure S12. Because of the intrinsic overlap of the conduction band and valence band, it shows a low PF as well as *zT*. It is also important to emphasize that other essential factors, such as thermal stability and mechanical integrity, must also be considered [8]. Furthermore, the ability of the TEiM materials to achieve high densification during synthesis is also a critical factor, as poor densification can lead to weak interfacial bonding, increased contact resistance, and mechanical failure during device operation. In Figure 3c, the measured density of the sample *x* = 0.05 is ∼ 5.18 g mm^−3^, which is significantly higher than ∼ 4.87 g mm^−3^ of the *x* = 0 sample. The Vickers hardness is also increased with 5% Ag doping, which proves higher densification obtained during the sintering process, exhibiting superior mechanical properties.

To check the *ρ*_c_ of the *x* = 0.05 sample, a MgAgSb‐based TE single leg is synthesized and measured. MgAg_0.97_Cu_0.03_Sb is sandwiched between MgAg_0.05_Cu_0.95_Sb and sintered into a three‐layer disk, as the optical photo of a single leg in Figure 3d. The *ρ*_c_ is measured by a home‐made four‐probe device and calculated based on the equation: *ρ*_c_ = *A* × *V*_jump_/*I* = *A* × *R*_c_, where *A* represents the cross section of the sample, *V*_jump_ represents the voltage jump between TEiM and TE material, *I* represents the test current, and *R*_c_ represents the contact resistance (Figure S13). The scan curve of resistance is presented in Figure 3d. Two distinct regions are identified in the MgAg_0.05_Cu_0.95_Sb TEiM and MgAg_0.97_Cu_0.03_Sb TE material in the probe distance dependence of resistance. Only a minimal resistance jump is detected across the interface between the TEiM and the TE material, and the value of *ρ*_c_ is observed to be about ∼6.27 µΩ cm^2^. A single leg using the sample with *x* = 0 as the TEiM was also synthesized to compare *ρ*_c_. As shown in Figure S14, a *ρ*_c_ value of ∼9.16 µΩ·cm^2^ is obtained, which is higher than that of the sample with *x* = 0.05 as the TEiM, suggesting better electronic contact in the sample *x* = 0.05. Considering the command of long‐term service of the fabricated single leg in the future, it is necessary to test its stability at working temperature. To simulate realistic service conditions, ambient‐air aging is chosen. Figure 3e shows the probe distance dependence of the resistivity at 573 K under various aging durations, and it can be observed that the contact resistivity remains relatively stable even after 20 days of aging, exhibiting the high potential of MgAg_0.05_Cu_0.95_Sb as TEiM for MgAg_0.97_Cu_0.03_Sb TE material.

### 2.4 MgAgSb/Mg_3_(Bi, Sb)_2_ Thermoelectric Module

**FIGURE 4:**
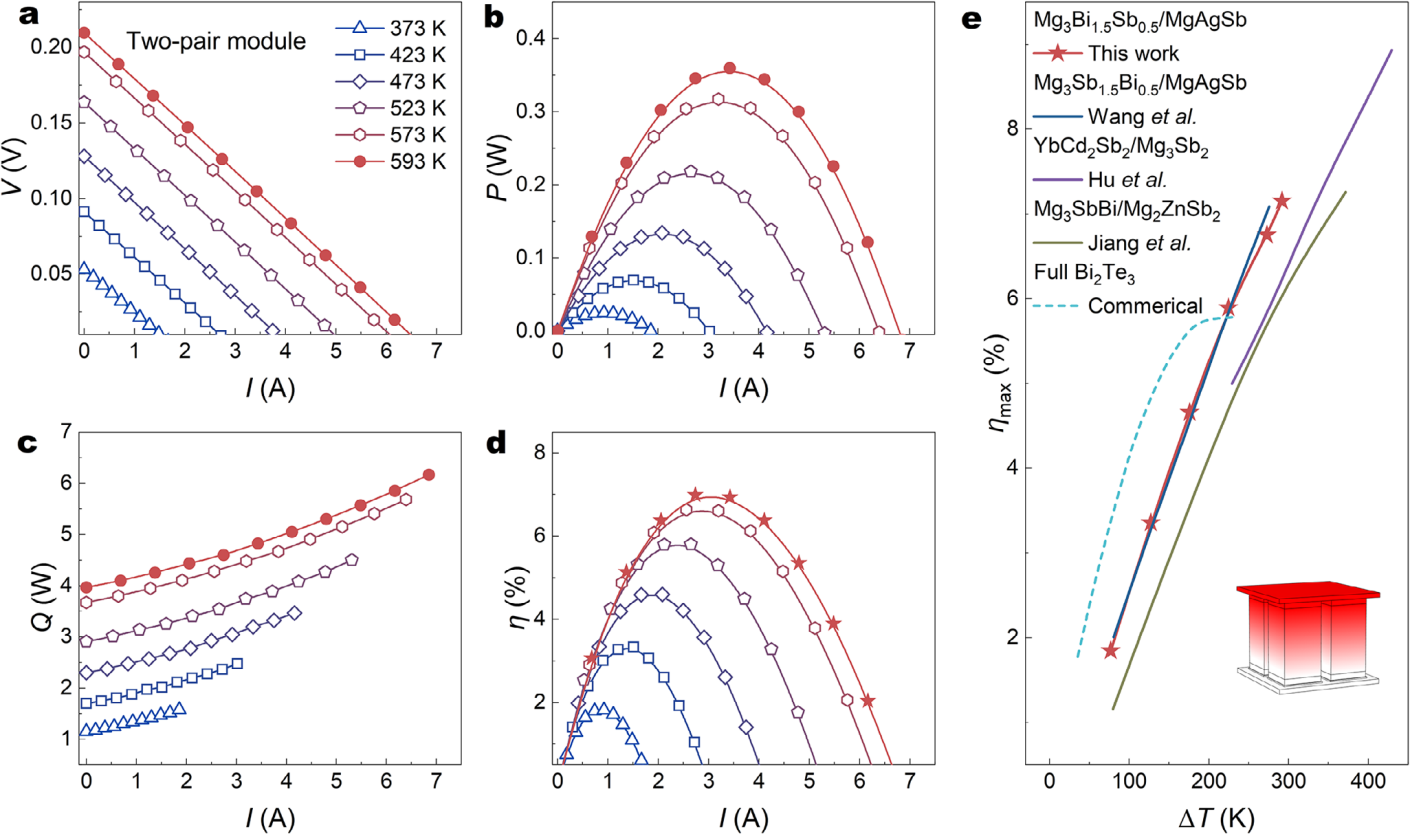
(a–c) The *I* dependence of (a) *V*, (b) *P*, (c) *Q*, and (d) *η* under different Δ*Ts* of the two‐pair TE module based on p‐type MgAg_0.97_Cu_0.03_Sb and n‐type Mg_3_(Sb, Bi)_2_, (e) Δ*T* dependence of *η_max_* in MgAgSb/Mg_3_(Sb, Bi)_2_ two‐pair module and its comparison with other reported two‐pair Mg‐based TE modules [52, 53, 54] as well as the commercial full Bi_2_Te_3_‐based device [55].

To directly evaluate the potential in low‐grade heat harvesting of our TE single leg, one two‐pair TE module using MgAg_0.97_Cu_0.03_Sb as p‐type legs and Mg_3_(Sb, Bi)_2_ as n‐type legs is fabricated and assembled. For the selection of n‐type legs, considering the high TE performance of Bi‐rich Mg_3_(Sb, Bi)_2_, Mg_3.2_Bi_1.5_Sb_0.45_Te_0.05_In_0.02_ is chosen as the n‐type material with the performance detailed in Figure S15 [50], which exhibits a notable *zT* ∼0.8 and ∼1.3 at 300 and 500 K, respectively. Optimized MgAg_0.05_Cu_0.95_Sb and conventional 304 stainless steel (304 SS) [51] are applied for the TEiM for p‐type and n‐type legs for maintaining the carrier transport and suppressing elements diffusion, as the optical image of TE modules and measurement set‐up in Figure S16. The output current (*I*), output voltage (*V*), output power (*P*), heat flow (*Q*), and conversion efficiency (*η*) are systemically characterized by a commercial apparatus, mini‐PEM, under different temperature gradients (*∆T*), and the largest hot‐side temperature (*T*_h_) is 593 K. The measured output voltage *V* is displayed in Figure 4a, which exhibits a good linear relationship. The internal resistance and open‐circuit voltage of the two‐pair module can be obtained. The reduced slope is consistent with the increased *σ* at high temperature. When this *T*_h_ is 593 K, the maximum output power *P*_max_ is around ∼0.36 W (Figure 4b), proving the high potential for power generation when the heat source is unlimited or free. Here, we also provide the comparison of maximum power density (*ω*_max_) since high *ω*_max_ directly determines the output power capability and overall energy utilization rate of TE devices, making it indispensable for real‐world applications. The value of *ω*_max_ is obtained based on *ω*_max_ = *P*_max_/*A*, where *A* represents the total cross‐sectional area of the TE device (10 mm × 10 mm). As a result, a high value of *ω*_max_ ∼0.36 W cm^−2^ is obtained under Δ*T* ≈293 K (Figure S17), highlighting its strong potential for practical application. Figure 4c shows the measure *Q* based on the temperature difference of the standard sample, which increases with *I* contributed from the Joule and Peltier heat. As a result, *η* is calculated based on the equation *η* = *P*/ (*P* + *Q*_out_), as the Figure 4d. The maximum conversion efficiency (*η*_max_) ∼7.2% is obtained under Δ*T* of 293 K (Figure 4e), which is comparable with other state‐of‐the‐art results [26, 47]. It also shows a comparable performance and high potential with other reported Mg‐based modules and the commercial Bi_2_Te_3_‐based TE unit [52, 53, 54, 55].

## 3 Conclusions

In summary, a systematic investigation of the MgAgSb‐MgCuSb binary system has been conducted to identify optimal compositions for TE applications, encompassing TEMs and TEiMs. XRD analysis reveals a phase transition from *α*‐MgAgSb to MgCuSb with an increase in the Cu content, accompanied by notable changes in electrical and thermal transport behavior. In the MgAg*_x_* Cu_1‐_ *_x_* Sb system, the Ag‐rich region (*x* = 0.95 to 1) displays relatively higher PF and *zT*, indicating its suitability as the TE material. The highest PF of ∼21 µW cm^−1^ K^−2^ at 323, as well as a competitive *zT* of 1.12 at 473 K, are obtained in MgAg_0.97_Cu_0.03_Sb, contributed from the increase in carrier concentration and suppression of the phonon transport. On the other side, the Cu‐rich region (*x* = 0 to 0.1) exhibits enhanced *σ* and *κ*, with the *x* = 0.05 sample demonstrating superior carrier and thermal transport capabilities compared to the *x* = 0 sample at elevated temperatures. These findings narrow down the ideal composition ranges of MgAgSb‐MgCuSb for TE junctions and highlight the potential of *x* = 0.97 and *x* = 0.05 as promising candidates for TEM and TEiM components, respectively. The corresponding 2‐pair modules coupled with n‐type Mg_3_(Sb, Bi)_2_ are fabricated, and a maximum efficiency of ∼7.2% is achieved under Δ*T* of 293 K. The proposed alloying strategy enables a holistic design of high‐performance TE modules, including TeiM development, and provides valuable insights for promoting the industrialization of Te‐free TE modules for power generation.

## 4 Experimental Section

### 4.1 Materials Preparation

High‐purity Mg turnings (99.95%, Sigma–Aldrich), Sb grains (99.999%, 5N Plus), Cu powders (≥99.9%, Sigma–Aldrich), and Ag powders (≥99.9%, Sigma–Aldrich) were weighed according to the nominal composition of MgAg*_x_* Cu_1‐_ *_x_* Sb (*x* = 0–1) inside a glovebox, where the oxygen and moisture levels were maintained below 1 ppm. For the sample MgAg*_x_* Cu_1‐_ *_x_* Sb with 0.5 wt.% C_18_H_36_O_2_ (x = 0.97, 0.99, and 1), C_18_H_36_O_2_ powder (99.9%) was also added during the weighing process. The weighed elements were then loaded into a stainless‐steel jar along with two stainless‐steel balls (12.5 mm in diameter) and subjected to high‐energy ball milling for 5 h. The resulting homogeneously mixed powders (∼2 grams per batch) were immediately transferred into a graphite die (10 mm inner diameter) and consolidated via spark plasma sintering (SPS) at 573 K under a uniaxial pressure of 60 MPa for 5 min to obtain high‐density disk‐shaped samples. The sintered disks were subsequently annealed in a muffle furnace at 573 K for 48 h in air, without applying vacuum protection.

### 4.2 Phase and Microstructure Characterizations

The phase structures of the sintered disk samples were characterized by X‐ray diffraction (XRD, SmartLab3, Rigaku) using Cu Kα radiation (λ ≈ 1.5406 Å), with a scanning range of 2θ = 10°–80° under continuous rotation mode. The microstructure and elemental composition of the SPS‐processed samples were examined using field emission scanning electron microscopy (FESEM, Hitachi SU8000), equipped with an energy‐dispersive X‐ray spectroscopy (EDX) detector for compositional analysis.

### 4.3 Material Property Characterizations

Bar samples cut from the pressed disks were used for electrical resistivity (*ρ*) and Seebeck coefficient (*S*) measurement via a commercial system (ULVAC ZEM‐2). The PF (Power Factor) was calculated based on the formula PF *= S^2^/ρ*. Total thermal conductivity *κ* was obtained according to *κ* = *DdC_p_*, where *D* is thermal diffusivity, *d* is the sample density, and *C*_p_ is the heat capacity at constant pressure. The density *d* of the samples was confirmed by the Archimedes method. Thermal diffusivity *D* and heat capacity *C*_p_ of the disk sample were measured simultaneously by a laser flash system (Netzsch LFA 467, Germany). Electronic thermal conductivity *κ*_e_ was calculated by the Wiedemann‐Franz law: *κ*_e_ = *LσT*, where *L* is the Lorenz number, which can be expressed by the equation: *L* = 1.5 + exp [‐|*S*|/116]. Lattice thermal conductivity *κ*_L_ was obtained by subtracting electronic thermal conductivity from the total thermal conductivity.

### 4.4 Single‐Leg and 2‐Pair Module Fabrication and Characterization

The MgAg_0.97_Cu_0.03_Sb single‐leg with MgAg_0.05_Cu_0.95_Sb contact layers at both ends was prepared by SPS: The ball‐milled MgAg_0.97_Cu_0.03_Sb powder 2.5 g was loaded between the MgAg_0.05_Cu_0.95_Sb powder 0.25 g each side in the graphite die and sintered by one‐step SPS using the conditions as of bulk material sintering described before. For the fabrication of a 2‐pair module, the Mg_3.2_In_0.02_Bi_1.4_Sb_0.595_Te_0.005_ TE material was used as a counterpart n‐type leg. The n‐type powder was sandwiched between the 304SS powder on each side and sintered using the same conditions as reported. The obtained sandwich‐structure joints were ground, polished, and then cut into bulk with dimensions of ∼3.7 × ∼3.7 × ∼7.1 mm. The 2‐pair module fabrication was similar to the previous report. It was fabricated by positioning the p‐type and n‐type TE lags on the ceramic substrate with dimensions of 10 mm × 10 mm × 0.6 mm. Liquid In‐Ga eutectic alloy was smeared between the legs and Cu interconnecting electrodes to reduce the electrical and thermal contact resistances. The conversion efficiency of the single‐leg and 2‐pair module was measured with hot‐side temperatures of 100°C, 200°C, 300°C, and 320°C, utilizing a commercial testing apparatus (Mini‐PEM, ADVANCE RIKO, Japan) [56]. The cold‐side temperature was maintained at 20–25°C.

## Author Contributions

J.L. contributed to the methodology, the sample preparations, characterization, writing, and revising the manuscript. A.L. contributed to the refinement of the methodology and the revision of the manuscript. L.W. contributed to the preparation of n‐type materials, the construction of the 2‐pair module, and the revision of the manuscript. X.W. contributed to the revision of the manuscript. T.M. led the project, providing supervision, conceptualization, methodology, writing, review and editing, and funding acquisition. All authors contributed to the review of the final manuscript.

## Conflicts of Interest

The authors declare no conflicts of interest.

## Supporting information




**Supporting File**: advs73838‐sup‐0001‐SuppMat.docx.

## Data Availability

The data that support the findings of this study are available in the supplementary material of this article.
