# Supplementary material for: Thermoelectric Property Mapping for High‐Performance Integrated MgAgSb‐MgCuSb System
Source: Adv Sci (Weinh). 2026 Jan 15;13(17):e20889. doi: 10.1002/advs.202520889 (PMC13042869; doi:10.1002/advs.202520889)
Supplement: Supplementary file 1 — Supporting File: advs73838‐sup‐0001‐SuppMat.docx. [file ADVS-13-e20889-s001.docx]

`

Supplement information

**Thermoelectric property mapping for high-performance integrated MgAgSb-MgCuSb system**

*Jiankang Li^1,2^, Airan Li^1^, Longquan Wang^1^, Xinzhi Wu^1^, Raju Chetty^1^, Takao Mori^1,2 *^*

^1^*Research Center for Materials Nanoarchitectonics (MANA), National Institute for Materials Science (NIMS), Namiki 1-1, Tsukuba 305-0044, Japan*

^2^*Graduate School of Pure and Applied Sciences, University of Tsukuba, Tennodai 1-1-1, Tsukuba 305–8671, Japan*

E-mail: MORI.Takao@nims.go.jp

**Figure S1.** the schematic diagram of one 2-pair TE module, which contains ceramic substrate, Cu electrode, TEiM, and TE materials.

**Figure S2.** The EDS mapping results of MgAgSb.

**Figure S3.** The EDS mapping results of MgAg_0.95_Cu_0.05_Sb. The nominal composition is also shown in the table.

**
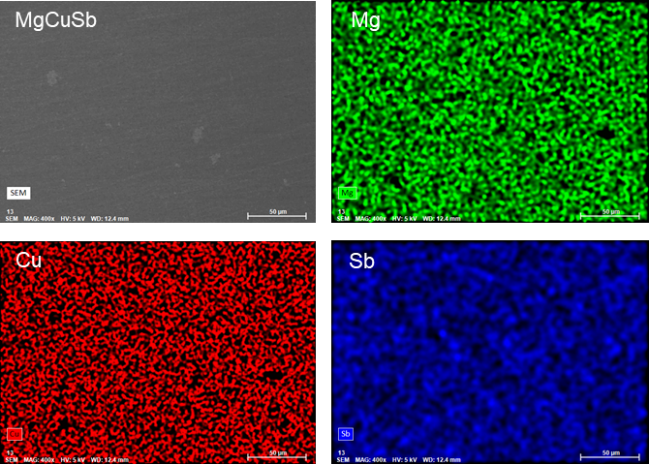
**

**Figure S4.** The EDS mapping results of MgCuSb.

**Figure S5.** Temperature-dependent (a) Seebeck coefficient, (b) power factor of MgAg*_x_*Cu_1-_*_x_*Sb (*x* = 0-1).

**Figure S6.** (a-b) Temperature-dependent (a) electronic thermal conductivity, (b) lattice thermal conductivity of MgAg*_x_*Cu_1-_*_x_*Sb (*x* = 0-1).


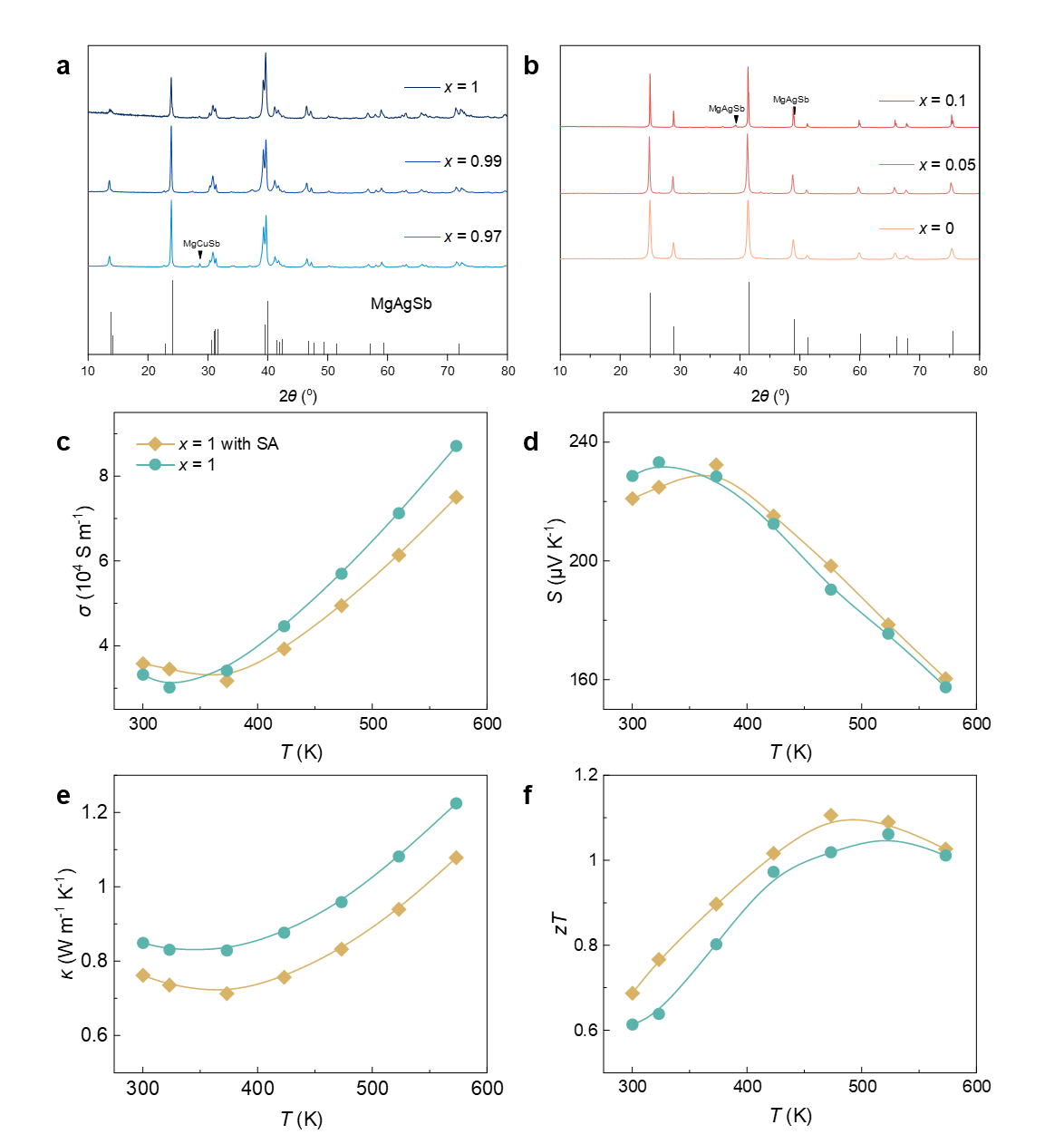


**Figure S7.** (a-b) XRD patterns of MgAg*_x_*Cu_1-_*_x_*Sb (a) *x* = 1, 0.99, and 0.97, (b) *x* = 0.1, 0.05, and 0. (c-f) Temperature-dependent (c) *σ*, (d) *S*, (e) *κ*, (f) *zT* of MgAg*_x_*Cu_1-_*_x_*Sb (*x* = 1) with/without the addition of steric acid.

**Figure S8.** The Rietveld refinement results of the MgAg*_x_*Cu_1-_*_x_*Sb (*x* = 0-1).


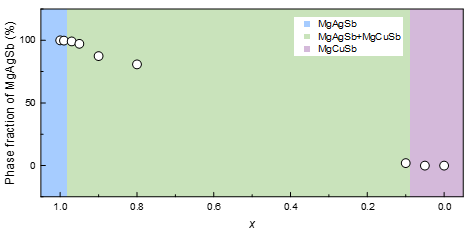

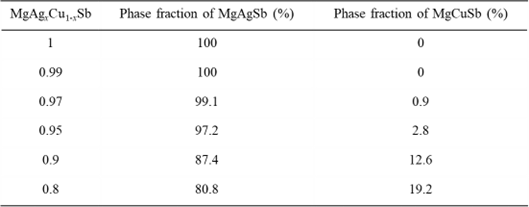


**Figure S9.** Room-temperature phase constitution of MgAg*_x_*Cu_1-_*_x_*Sb samples, showing the transition from single-phase MgAgSb to a two-phase mixture of MgAgSb and MgCuSb with increasing Cu content.

**Figure S10** EDS image of polished MgAg_0.97_Cu_0.03_Sb sample, where the distribution of Cu is homogeneous.


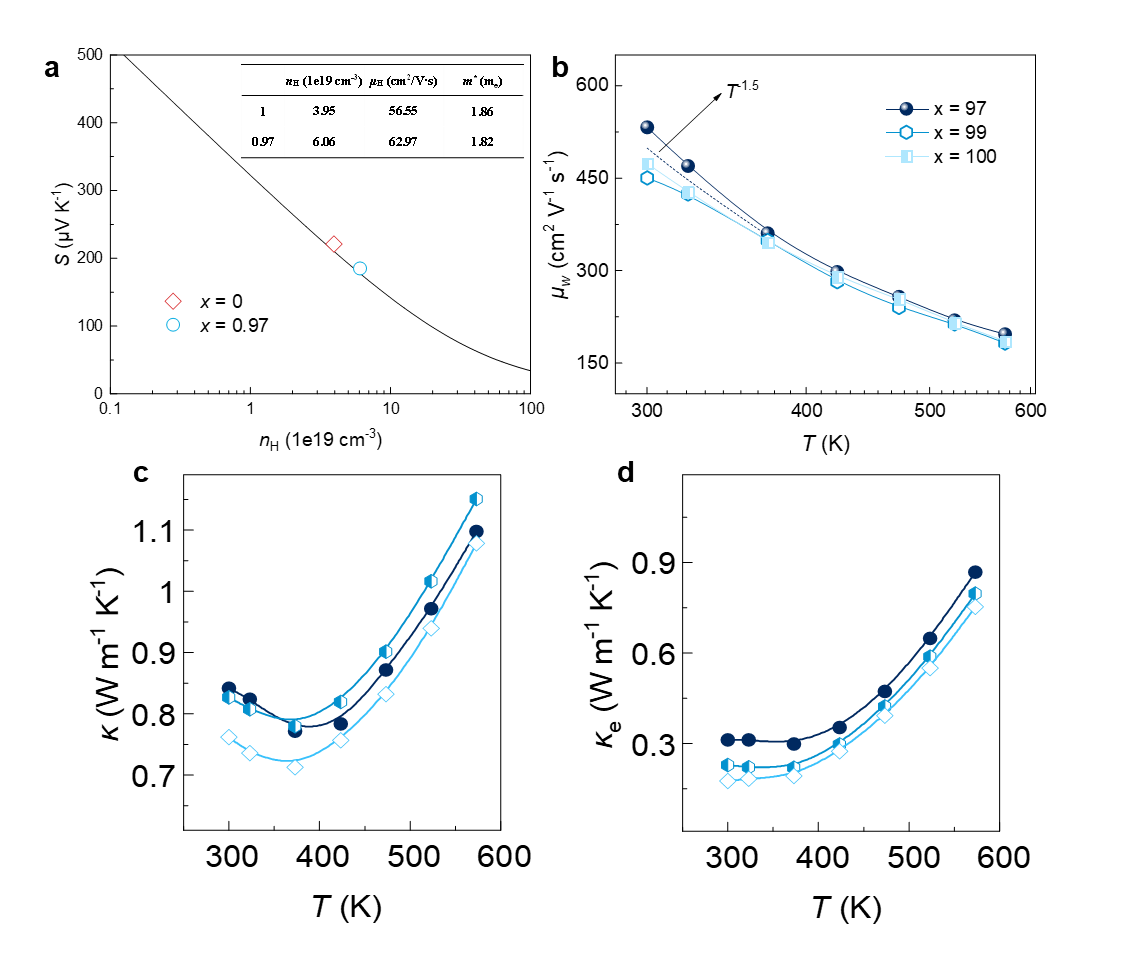


**Figure S11.** (a) The Pisarenko plot of MgAg*_x_*Cu_1-_*_x_*Sb (*x* = 0.97 and 1). (c-d) Temperature-dependent (b) *μ*_w_, (c) *κ*, (d) *κ*_e_ of MgAg*_x_*Cu_1-_*_x_*Sb (*x* = 0.97, 0.99 and 1).

**Figure S12.** (a-c) Temperature-dependent (a) Seebeck coefficient, (b) power factor, (c) *zT* of MgAg*_x_*Cu_1-_*_x_*Sb (*x* = 0, 0.05).

**Figure S13.** The sketch map of the Electrical Contact Resistivity measurement system.

**Figure S14.** Probe distance dependence of resistance in MgCuSb/MgAg_0.97_Cu_0.03_Sb TE single leg.

**Figure S15.** Temperature dependent (a) *σ*, (b) *S*, (c) *κ*, (d) *ZT* of Mg_3.2_In_0.02_Bi_1.4_Sb_0.595_Te_0.005_.


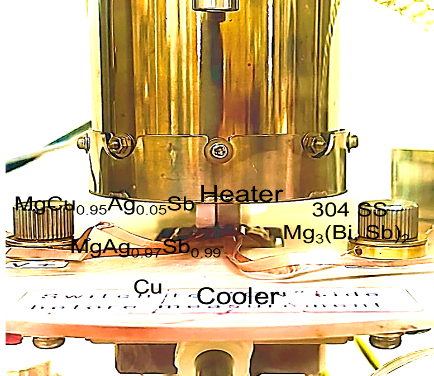


**Figure S16.** the optical image of TE modules and measurement set-up.


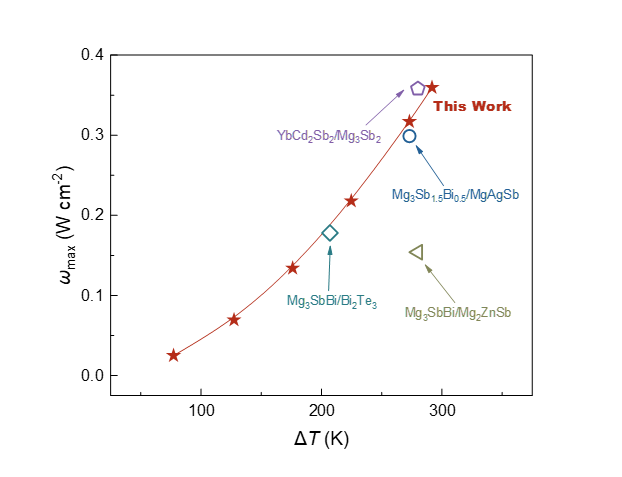


**Figure S17.** The maximum output power density (*ω*_max_) of our module under different Δ*T* and literature data of other TE modules are presented for comparison ^[1–4]^.

**Reference**

[1] L. Wang, W. Zhang, S. Y. Back, N. Kawamoto, D. H. Nguyen, T. Mori, *Nat. Commun.* **2024**, *15*, 6800.

[2] J. Lei, K. Zhao, J. Liao, S. Yang, Z. Zhang, T.-R. Wei, P. Qiu, M. Zhu, L. Chen, X. Shi, *Nat. Commun.* **2024**, *15*, 6588.

[3] J. Hu, Y. Sun, W. Shi, H. Wu, J. Zhu, J. Cheng, L. Jiao, X. Jiang, L. Xie, N. Qu, F. Li, Z. Yu, Q. Zhang, Z. Liu, F. Guo, W. Cai, J. Sui, *Adv. Mater.* **2024**, *36*, 2411738.

[4] M. Jiang, Y. Fu, Q. Zhang, Z. Hu, A. Huang, S. Wang, L. Wang, W. Jiang, *Natl. Sci. Rev.* **2023**, *10*, nwad095.
